# Analysis of Blood Glucose Distribution Characteristics and Its Risk Factors among a Health Examination Population in Wuhu (China)

**DOI:** 10.3390/ijerph13040392

**Published:** 2016-03-31

**Authors:** Jiangen Song, Xiaojuan Zha, Haibo Li, Rui Guo, Yu Zhu, Yufeng Wen

**Affiliations:** 1 School of Public Health, Wannan Medical College, Wuhu 241002, Anhui, China; songjian_gen@163.com (J.S.); haiboli89@163.com (H.L.); guorui0710075@126.com (R.G.); kutuomonk@foxmail.com (Y.Z.); 2 Department of Physical Examination, Yijishan Hospital of Wannan Medical College, Wuhu 241002, Anhui, China; 13705533568@139.com

**Keywords:** blood glucose, hyperglycaemia, impaired fasting glucose, diabetes, risk factors

## Abstract

*Background*: Diabetes mellitus (DM) and Impaired Fasting Glucose (IFG) represent serious threats to human health, and as a result, this study was aimed at understanding the blood glucose distribution characteristics and the risk factors among a large health examination population in China. *Methods*: An investigation with physical and biochemical examinations and questionnaires was conducted in the physical examination center from 2011 to 2014 and as a result 175,122 physical examination attendees were enrolled in this study. Multivariate logistic regression was used to explore the factors influencing blood sugar levels. *Results*: The rates of IFG and DM were 6.0% and 3.8%. Prevalence were 7.6%/5.1% in males and 5.1%/2.8% in females for IFG and DM, respectively. The prevalence of IFG and DM were thus higher in males than in females. In the normal group, except high density lipoprotein (HDL) that was significantly higher than in the IFG and DM group, the other indexes (age, body mass index (BMI), glucose (Glu), total cholesterol (TC) and total glycerides (TG) were lower than those in the IFG and DM group. The proportion of IFG and DM also increased with the increases in proportion of abnormal blood pressure, smoking and alcohol consumption. Multivariate logistic regression analysis showed that increasing age, high BMI, high TC, high TG and low HDL increased the risk of diabetes, while in males, in addition to the above factors, the smoking and drinking factors also increased the risk of diabetes. After the age of 65, the blood glucose level reached a peak in males, while in females, the increasing trends was on the rise. The inflexion age of the fast rise was younger in males than in females. *Conclusion*: The study population showed a high prevalence of DM and IFG among the adults. Regular physical examination for the early detection of diabetes is recommended in the high-risk population.

## 1. Introduction

Diabetes mellitus (DM) is a chronic systemic metabolic disease, which not only seriously harms human health, but causes a great socioeconomic burden [1,2]. The estimated prevalence of diabetes was 11.6% according to a national survey in China [1]. Along with the improvement of people’s economic level and the change of lifestyle, the incidence of DM is expected to increase to 12.1% by 2030. Similarly, the prevalence of DM will increase in other countries by 2030, such as the U.S. (11.8%), India (9.9%) and Australia (9.3%) [3]. More and more studies indicate that the DM has become a significant global public health problem [4,5]. Early detection, diagnosis and treatment are essential for the prevention and control of DM. Regular physical examination is an important way for early detection of DM and glucose intolerance. However DM is not noted in the general population. Many studies have found that the risk of DM would the reduced significantly if interventions or healthy lifestyles can be targeted at the abnormal group [6,7]. Published reports show variations in the risk factors and preventive strategies for DM. Diabetes risk decreased by 10% for each percentage point of weight loss with an intensive lifestyle intervention [7]. Lin [8] and Qi [9] reported that the level of blood glucose would decrease in the high-risk population undergoing intensive lifestyle intervention. However, objective measurement is lacking on the efficacy of interventions for diabetes, and at which point a given intervention would be effective in the high-risk population should be studied. An insight into the extent of the characteristic and risk factors for abnormal blood glucose in the general population is crucial for effective intervention. The results of this study may help to assess the socioeconomic burden of DM and encourage active screening and promotion of the timely control of diabetes among the general population in China.

## 2. Methods

### 2.1. Study Population

This study was performed by a cross-sectional physical examination method at Yijishan Hospital of Wannan Medical College, which is located in Wuhu City in China. The total sample, which included 175,122 cases ≥18 years old examined during 2011–2014, included 102,261 males (mean age 42.8 ± 13.0) and 72,861 females (age 40.4 ± 13.6).

### 2.2. Data Collection Procedure

This study was conducted in accordance with the Declaration of Helsinki and was approved by Ethics Committee of Wannan Medical College (2010wy038). Verbal informed consent was obtained from each subject before the investigation, all participants (volunteers aged 18 years or more) were identified. All the participants were requested to complete the questionnaire, physical examination and laboratory test. Participants’ demographic characteristics such as age and sex, and lifestyle habits such as smoking, and alcohol consumption were assessed by trained investigators. On the day of the survey, investigators explained the purpose of the study, the elements of informed consent, and distributed the survey. After that, participants were asked to complete questionnaires by themselves or with the help of the investigators. The completed questionnaires were checked by reviewers.

The questionnaires were followed by a general physical examination, which included a fasting blood collection, liver and kidney ultrasonography, and anthropometric assessment. The anthropometric assessments included height, weight and blood pressure. The weight was collected using a calibrated electronic digital scale, accurate to 100 g with subjects wearing only light underwear. Heights were measured to the nearest 0.1 cm using a secure portable stadiometer while wearing no shoes. The blood pressure was recorded twice by an experienced nurse in both arms with a mercury sphygmomanometer in the sitting position after a 5 min rest.

Also, blood samples were withdrawn from the antecubital vein of subjects in fasting status. High density lipoprotein (HDL), low density lipoprotein (LDL), triglyceride (TG), and total cholesterol (TC) were assessed by the clinical laboratory of the hospital. All laboratory values were assayed by an automated biochemical analysis instrument (DPP-800, Roche Pharmaceutical Ltd., Shanghai, China), using commercially available assays.

### 2.3. Study Definitions

In our study, glucose intolerance, DM and hypertension were considered. Diabetes mellitus was diagnosed according to the 1999 World Health Organization diagnostic criteria or previously diagnosed diabetes [10]: impaired glucose tolerance (FPG: 6.1–6.9 mmol/L); undiagnosed diabetes (FPG ≥ 7.0 mmol/L).

The diagnosis of hypertension was based on standard criteria according to the Guidelines for Prevention and Treatment of Hypertension in China [11]: Normal BP (SBP < 120 mm Hg and diastolic BP (DBP) < 80 mm Hg); Pre-HTN (these are patients on the cusp of developing HTN. It is defined as a SBP of 120–139 mm Hg or a DBP of 80–89 mm Hg); Stage I HTN (SBP 140–159 mm Hg or DBP 90–99 mm Hg); Stage II HTN (SBP ≥ 160 mm Hg or DBP ≥ 100 mm Hg); Stage III HTN (SBP ≥ 180 mm Hg or DBP ≥ 110 mm Hg). High TC was defined as a serum level of TC > 6.22 mmol/L. High TG was defined as a serum level of triglyceride ≥ 2.26 mmol/L. Low HDL-C was defined as a serum level of HDL-C < 1.04 mmol/L. Smoking or drinking was defined as smoking or drinking once a week, past behavior was considered to be no. “No” referred to no more than once a week or never, “Occasionally” referred to no more than three times a week, “Frequently” referred to more than five times a week.

### 2.4. Data Analysis

All participant information and laboratory results were entered into a customized database, and then analyzed by the R 3.0 statistical software (Chinese Academy of Sciences, Beijing, China). Descriptive analysis and chi-square tests were conducted to analyze the general characteristics of participants regarding lifestyle habits and glucose levels. The differences between laboratory results and lifestyle habits among different glucose level groups were compared using *t*-test or Chi squared, accordingly. Multivariate logistic regression analysis was used to estimate the risk factors for hyperglycemia (hyperglycemia status as a dependent variable; gender, age, BMI, TC, HDL-C, smoking status, drinking status as independent variables). The odds ratio (OR) and its 95% confidence interval (CI) were presented to show the risks. The trend in glucose with age for the different sexes was shown with a graphic. A *p*-value < 0.05 was considered as significant.

## 3. Results

### 3.1. Prevalence of DM and IFG in Different Sexes

A total of 175,122 people were evaluated in this study, among which 72,861 were female with a mean age of 40.4 ± 13.6 years. The prevalence of frequent alcohol consumption, smoking, Normal, IFG and DM among participants were 12.9%, 24.2%, 80.8%, 6.0% and 3.8%, respectively, and males showed a significantly higher prevalence than females (*p* < 0.001) (Table 1).

### 3.2. Characteristics of IFG and DM

As Table 2 shows, participants’ age, BMI, Glu, HDL, TC and TG were significantly different between different levels of plasma glucose (*p* < 0.001). The HDL of the normal group was higher than that of the IFG and DM groups, other variables were lower. An increasing rate of IFG and DM was associated with the increasing rate of abnormal BP, smoking and alcohol consumption.

### 3.3. Risk Factors of Hyperglycaemia

The level of blood glucose data was assigned to two groups: hyperglycemia (includes IFG and DM) and control. In a multivariate logistics analysis, using an enter method, a significant positive correlation was observed between blood glucose and participants’ age, BMI, BP, TG and TC as well as inverse correlations with HDL (Table 3 and Table 4). Concomitantly, smoking and alcohol use were associated with an increased risk of diabetes in males (Table 4).

### 3.4. The Trends of Glucose as Age

**Figure 1:**
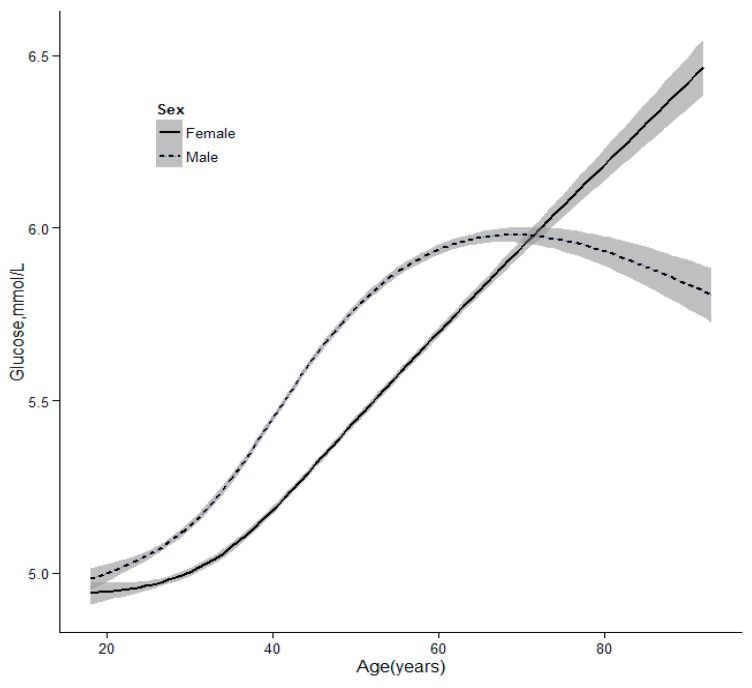
The trends of glucose levels with age according to sex.

As Figure 1 shows, there was a strong increasing tendency of glucose level with age. The glucose of males reached a peak at the age of 65, however, it continued to increase in female. The increasing inflection point of glucose in males was younger than for females. The level of FPG was higher in males than in females before 70, but the reverse was true after 70.

## 4. Discussion

This study was conducted among a 175,122 person physical examination population aged 18–95 from Wuhu City and the surrounding areas during 2011–2014. This large sample data only reflected the level of FPG and incidence of DM in the local population. However, a diagnosis of diabetes should not be excluded merely on the basis of glucose levels because this study included only one FPG result in this physical examination population, therefore the rate of undiagnosed diabetes was an outcome measured in our study.

Compared with the global prevalence of DM (2.8%), the local DM rate was higher (3.8%). Previous studies showed that the prevalence of DM was 2.8% in 2000, which would be expected to rise to 4.4% by 2030, and the number of DM cases would reach 5 million [3,6,7,12]. However, the rate of prevalence of DM was much higher than the national result in 2010 which was 2.2%. This might be explained by the rapid socioeconomic development of different dietary habits. A study in 2011 reported a prevalence of 10.1% for DM in the city of Shanghai [13]. Another cross-sectional survey among 3919 subjects found the prevalence of DM and IFG were 5.9% and 10.0% [14]. Also, the prevalence of DM was somewhat different in different areas. This might be due to different economic levels, the age structure of the population, geographical distribution of people and dietary habits and so on.

The current study showed that the prevalence of IFG was positively correlated with age, which was similar to previous studies [15,16,17]. The possible reason is that oxidative phosphorylation dysfunction of islet cells, with a decrease in the amount and secretory functions, result in glucose increases with age [18]. Furthermore, this study showed a significant difference in the frequency of DM between males (5.1%) and females (2.8%). Some studies found a similar result in that the incidence of DM was higher in men than women but not others [19,20,21]. Habits such as smoking, alcohol consumption and physical activity levels contribute to the increase of DM in males. In addition, the results were similar to previous studies [8,13,22,23], where increasing age, high BMI, high TC, high TG and low HDL increased the risk of diabetes. The process of glucose metabolism was strongly correlated with factors such as BMI, BP and TC. Also, the development and progression of DM interacts with related disease such as obesity, hypertension and hyperlipidemia. It is noteworthy that smokers and drinkers would show increased risk of diabetes compared to non-smokers and non-drinkers in males. The results can help target certain groups for DM educational initiatives, especially smokers and drinkers, so as to appropriately reduce the risk of DM in this high-risk population.

As Figure 1 shows, there was a strong increasing tendency of glucose with age. The glucose of males reached a peak at the age of 65, while it always continued increasing in females. An apparent sex and age difference in the blood glucose levels was found because of differences in physiology of sex, levels of the hormones across age and related factors [20,24,25]. In China, life and job stress are much greater in males than in females before retirement, so little attention has been paid to their health. Also, an unhealthy lifestyle may lead to a rise in blood glucose. In contrast, many efforts have focused on the health of males such as physical exercise, which may lead to a decrease in blood glucose concentration [26,27]. However, the decline in circulating estrogen concentration after the menopause is associated with metabolic imbalances of glucose. Maybe these factors explain the differences among different genders before or after retirement. The result indicates that the blood glucose of males should be monitored at a younger age. Although DM is often associated with a poor prognosis, earlier detection may improve outcomes.

## 5. Limitations

One of the major advantages of this study is that it was a large sample investigation, with subjects from the same area, which support the accuracy and stability of the result. Of course, the present study has some limitations. First, FPG but not has oral glucose tolerance test (OGTT) been used as an indicator of blood glucose because of the specificity and acceptability in physical examination groups. In general, OGTT shows higher sensitivity and specificity for diagnosis of diabetes. More studies are needed to confirm these findings in the future. Second, some questions in the questionnaire are mainly subjective and the fact the results were self-reported may lead to bias. Finally, our study failed to explore the causality relationship between blood glucose and related factors because of inherent limitations of the cross-sectional study. Despite these limitations, the results of this study are noteworthy for DM education and intervention.

## 6. Conclusions

In summary, high prevalence of DM and IFG was observed in a large population of adults. Various factors such as age, sex and other disorders contribute to hyperglycemia, especially lifestyle habits. It is particularly important to strengthen community care of DM, screen high-risk groups and adopt appropriate preventive measures.

## Figures and Tables

**Table 1:** Frequency of different variables related to the participants.

Variable	Total (*n* = 175,122)	Females (*n* = 72,861)	Male (*n* = 102,261)	*t*/χ^2^	*p*
Age	40.1 ± 13.9	40.4 ± 13.6	42.8 ± 13.0	7.8	<0.001
Alcohol				49,495.9	<0.001
No	97,297 (63.8)	58,747 (97.7)	38,550 (41.8)		
Small	28,188 (18.5)	597 (1.0)	27,591 (29.9)		
Occasionally	7231 (4.7)	619 (1.0)	6612 (7.2)		
Frequently	19,740 (12.9)	167 (0.3)	19,573 (21.2)		
Smoking				42,879.1	<0.001
No	105,542 (69.2)	59,864 (99.6)	45,678 (49.5)		
Small	7401 (4.9)	63 (0.1)	7338 (7.9)		
Occasionally	2683 (1.8)	27 (0.0)	2656 (2.9)		
Frequently	36,829 (24.2)	175 (0.3)	36,654 (39.7)		
Glucose *				922.7	<0.001
Normal	141,509 (80.8)	58,089 (92.1)	83,420 (87.3)		
IFG	10,487 (6.0)	3193 (5.1)	7294 (7.6)		
DM	6580 (3.8)	1779 (2.8)	4801 (5.1)		

*: The glucose levels of 16,546 study participants were not collected.

**Table 2:** The characteristic of participants between different plasma glucose levels.

Variable	Normal (*n* = 141,509)	IFG (*n* = 10,487)	DM (*n* = 6580)	F/χ^2^	*p*
Age (years)	39.52 ± 13.37	50.47 ± 12.99	53.75 ± 12.00	6552.0	<0.001
BMI (kg/m^2^)	23.36 ± 3.38	25.18 ± 3.30	25.66 ± 3.29	2518.2	<0.001
Glu (mmol/L)	5.12 ± 0.45	6.42 ± 0.24	9.26 ± 2.72	122,183.0	<0.001
HDL (mmol/L)	1.33 ± 0.36	1.30± 0.36	1.25 ± 0.34	172.7	<0.001
TC (mmol/L)	4.60 ± 0.86	4.95 ± 0.91	5.04 ± 1.03	1367	<0.001
TG (mmol/L)	1.47 ± 1.16	1.94 ± 1.64	2.38 ± 2.40	2016	<0.001
BP				7375.1	<0.001
Normal	58,046 (95.5)	1803 (3.0)	935 (1.5)		
Pre-HTN	57,948 (88.8)	4605 (7.1)	2714 (4.2)		
Stage I	14,327 (77.6)	2427 (13.1)	1711 (9.3)		
Stage II	4972 (73.7)	1002 (14.9)	769 (11.4)		
Stage III	919 (70.7)	225 (17.3)	156 (12.0)		
Alcohol				997.9	<0.001
No	85,911 (90.6)	5478 (5.8)	3448 (3.6)		
Small	24,214 (87.5)	2156 (7.8)	1301 (4.7)		
Occasionally	6497 (92.6)	343 (4.9)	173 (2.5)		
Frequently	16,172 (83.6)	1913 (9.9)	1261 (6.5)		
Smoking				309.0	<0.001
No	92,490 (89.8)	6633 (6.4)	3857 (3.7)		
Small	6479 (89.4)	506 (7.0)	264 (3.6)		
Occasionally	2408 (93.1)	117 (4.5)	61 (2.4)		
Frequently	31,419 (87.2)	2632 (7.3)	2000 (5.5)		

**Table 3:** The factors of glucose on the logistic regression analysis in females.

Variable	β	SE	Beta	OR	95% CI	*p*
Age (years)	0.06	0.00	4.72	1.07	1.06–1.07	<0.001
BMI (kg/m^2^)	0.08	0.01	1.44	1.08	1.07–1.10	<0.001
TG (mmol/L)	0.18	0.02	0.93	1.19	1.15–1.24	<0.001
TC (mmol/L)	0.16	0.03	0.84	1.17	1.1–1.25	<0.001
HDL (mmol/L)	−0.61	0.09	−1.28	0.54	0.45–0.65	<0.001
BP						
Normal	0	-		1	-	
Pre-HTN	0.50	0.07		1.65	1.43–1.91	<0.001
Stage I	0.80	0.09		2.23	1.89–2.64	<0.001
Stage II	0.92	0.11		2.51	2.01–3.13	<0.001
Stage III	1.15	0.23		3.17	2.02–4.97	<0.001

**Table 4:** The factors of glucose on the logistic regression analysis in males.

Variable	β	SE	Beta	OR	95% CI	*p*
Age (years)	0.06	0.00	3.16	1.06	1.06–1.06	<0.001
BMI (kg/m^2^)	0.09	0.01	1.22	1.09	1.08–1.10	<0.001
TG (mmol/L)	0.13	0.01	0.85	1.14	1.12–1.16	<0.001
TC (mmol/L)	0.23	0.02	0.86	1.26	1.21–1.31	<0.001
HDL (mmol/L)	−0.50	0.06	-0.67	0.61	0.54–0.69	<0.001
BP						
Normal	0	-		1	-	
Pre-HTN	0.29	0.05		1.33	1.21–1.47	<0.001
Stage I	0.56	0.06		1.76	1.58–1.96	<0.001
Stage II	0.71	0.07		2.03	1.79–2.31	<0.001
Stage III	0.65	0.11		1.92	1.55–2.40	<0.001
Smoking						
No	0	-		1	-	
Small/Occasionally	−0.11	0.07		0.89	0.78–1.02	0.085
Frequently	0.14	0.04		1.15	1.07–1.24	<0.001
Alcohol						
No	0	-		1	-	
Small/Occasionally	−0.01	0.04		0.99	0.91–1.07	0.725
Frequently	0.16	0.04		1.17	1.07–1.28	0.001

## Abbreviations

The following abbreviations are used in this manuscript:

DM: diabetes mellitus
FPG: impaired glucose tolerance
HDL: high density lipoprotein
LDL: low density lipoprotein
TG: triglyceride
TC: total cholesterol
OGTT: oral glucose tolerance test
